# Perspective in Alternative Splicing Coupled to Nonsense-Mediated mRNA Decay

**DOI:** 10.3390/ijms21249424

**Published:** 2020-12-10

**Authors:** Juan F. García-Moreno, Luísa Romão

**Affiliations:** 1 Department of Human Genetics, Instituto Nacional de Saúde Doutor Ricardo Jorge, 1649-016 Lisboa, Portugal; juan.moreno@insa.min-saude.pt; 2 Faculty of Science, BioISI—Biosystems and Integrative Sciences Institute, University of Lisboa, 1749-016 Lisboa, Portugal

**Keywords:** alternative splicing (AS), nonsense-mediated RNA decay (NMD), AS-NMD, gene expression regulation

## Abstract

Alternative splicing (AS) of precursor mRNA (pre-mRNA) is a cellular post-transcriptional process that generates protein isoform diversity. Nonsense-mediated RNA decay (NMD) is an mRNA surveillance pathway that recognizes and selectively degrades transcripts containing premature translation-termination codons (PTCs), thereby preventing the production of truncated proteins. Nevertheless, NMD also fine-tunes the gene expression of physiological mRNAs encoding full-length proteins. Interestingly, around one third of all AS events results in PTC-containing transcripts that undergo NMD. Numerous studies have reported a coordinated action between AS and NMD, in order to regulate the expression of several genes, especially those coding for RNA-binding proteins (RBPs). This coupling of AS to NMD (AS-NMD) is considered a gene expression tool that controls the ratio of productive to unproductive mRNA isoforms, ultimately degrading PTC-containing non-functional mRNAs. In this review, we focus on the mechanisms underlying AS-NMD, and how this regulatory process is able to control the homeostatic expression of numerous RBPs, including splicing factors, through auto- and cross-regulatory feedback loops. Furthermore, we discuss the importance of AS-NMD in the regulation of biological processes, such as cell differentiation. Finally, we analyze interesting recent data on the relevance of AS-NMD to human health, covering its potential roles in cancer and other disorders.

## 1. Introduction

The mRNA in eukaryotic cells undergoes a variety of processes from gene transcription to mRNA translation and degradation. Three major pre-mRNA modifications occur co-transcriptionally in the nucleus: 5′ capping and addition of a 3′ poly(A) tail, both enhancing mRNA stability and facilitating translation, and pre-mRNA splicing, where introns are removed from the primary transcript. During splicing, exons can be joined through distinct combinations in a process known as alternative splicing (AS), generating different transcripts produced from a single gene. In most cases, the resulting multiple transcript isoforms are translated into different proteins with distinct properties [1,2]. Once this pre-mRNA processing stage is concluded, the mature mRNA is exported to the cytoplasm along with several associated proteins, many of them acquired during pre-mRNA processing, forming a messenger ribonucleoprotein particle (mRNP). This mRNP complex allows the cell to inspect the mRNA before its export to the cytoplasm, in order to avoid processing defects [3]. In the cytoplasm, there is a switch of some mRNP factors to facilitate translation initiation. To ensure a proper protein synthesis, several cytoplasmic surveillance mechanisms, such as the nonsense-mediated RNA decay (NMD) pathway, control the mRNA quality. NMD selectively degrades mRNAs harboring premature translation-termination codons (PTCs), thus reducing the expression of truncated proteins.

During the past decade, multiple examples of coordinated action between AS and NMD have been reported across several species, arising as a new post-transcriptional instrument of gene expression regulation in the cell. It is estimated that AS gives rise to >80,000 protein-coding transcripts from the human genome, which includes less than 20,000 genes, according to the GENCODE project (version 35 (https://www.gencodegenes.org/human/stats.html)). Interestingly, there are also more than 16,000 transcripts annotated as NMD targets, most of them resulting from AS. AS coupled to NMD (AS-NMD) fine-tunes the expression of multiple transcripts, having an important role in shaping the transcriptome. This paper covers the last discoveries made in the field, summarizing the mechanisms behind AS-NMD, as well as its biological relevance in tissue-specific gene expression regulation. We also describe examples of human diseases associated with AS-NMD dysregulation and discuss the implication of these findings for diagnosis and treatment.

## 2. Pre-mRNA Splicing

Pre-mRNA splicing is a crucial process in eukaryotic cells, which occurs either during or after transcription in the nucleus [4]. It allows the removal of introns, which are within the pre-mRNA and the concomitant joining of coding sequences, in a complex process coordinated by the spliceosome [1,2]. The spliceosome is a dynamic multi-megadalton ribonucleoprotein complex, assembled by five small nuclear ribonucleoproteins (snRNPs) (U1, U2, and U4-U6) and numerous auxiliary proteins [5]. Pre-mRNA splicing is determined by the 5′ and 3′ splice sites and the branch point site [6], which usually are short and weakly conserved intronic sequences that define where the introns are spliced out through transesterification reactions [1]. Initially, the U1 snRNP is recruited to the 5′ splice site together with the splicing factor U2AF. Next, U2 snRNP associates with the branch point, resulting in the A complex, also known as pre-spliceosome, which defines exon and intron boundaries. Then, the U4/U6.U5 tri-snRNP is recruited (B complex) and major RNA-RNA and RNA-protein rearrangements lead to the formation of the active spliceosomal C complex that catalyzes the ligation of two exons [5]. Despite the splicing mechanism having been extensively studied, further research is needed to identify which are the consensus sequences that facilitate splice site selection and allow the accurate recognition of exons.

Splicing is regulated through *cis*-acting elements, such as intronic splicing enhancers (ISEs) or silencers (ISSs), and exonic splicing enhancers (ESEs) or silencers (ESSs). Such *cis*-acting elements are usually short and diverse sequences [5] that function as motifs for interaction with *trans*-acting RNA-binding proteins (RBPs), mainly heterogeneous nuclear ribonucleoproteins (hnRNPs) [7,8,9] and serine arginine-rich (SR) proteins [10,11]. Most hnRNPs, such as hnRNP A/H, or the polypyrimidine tract-binding protein (PTB) function as splicing repressors, since they promote exon skipping by altering the 5′ and 3′ splice site choice [7,12,13,14,15]. On the other hand, SR proteins usually bind to ESEs to facilitate the recruitment of the spliceosomal complex U1 snRNP to the 5′ splice site, thus activating splicing. However, given that the regulatory functions of these *trans*-acting factors are position-dependent and influenced by the surrounding RBPs, in some cases, hnRNPs can activate exon inclusion [16,17], and SR proteins can act as repressors. For instance, SR proteins can bind to ISS regions and consequently repress splicing, as reported in several studies [18,19,20,21]. These data suggest that the repressive and enhancer functions of splicing factors are highly dependent on their binding locations.

## 3. Alternative Pre-mRNA Splicing

While some exons are constitutively incorporated in the mature mRNA, many others are alternatively spliced, so different mRNA versions can be generated from a single pre-mRNA by AS. Therefore, from one single gene locus, AS allows the creation of different alternative spliced RNAs (AS RNAs), some of them translated into proteins that may have distinct functions and/or locations in the cell. AS is estimated to occur in around 95% of multi-exon genes [22,23], representing a source of high proteomic diversity [24]. In addition to generating functionally distinct protein isoforms, AS also controls gene expression levels. This is accomplished through different mechanisms, for example, by generating PTC-containing isoforms, and committing them to NMD, as explained in more detail in the following sections. Additionally, AS can generate alternative 5′ and 3′ untranslated regions (UTRs), which impact translation efficiency, mRNA stability and localization in the cytoplasm [25]. Advanced technologies such as genome-wide approaches have allowed the identification of numerous biological processes where AS plays a crucial regulatory role, such as stem cell pluripotency maintenance and cell differentiation [26,27,28,29], neural development [30], cell survival [31], membrane-trafficking [32], immune system [33,34], and cell proliferation [35,36], etc.

Several modes of AS have been described. The most prevalent ones in mammals are cassette exon, where the exon is either included or skipped out from the transcript (exon inclusion or skipping, respectively), and usage of alternative 5′ and 3′ splice sites, in which shorter or longer versions of an exon are spliced [23,37]. Other important AS patterns consist of mutually exclusive exons, where just one of two exons can be included in the mRNA isoform, and intron retention, when there is no intron excision. AS can also establish patterns, such as alternative polyadenylation, that do not produce alterations in the coding sequences, but can deeply affect the mRNA fate. [38,39].

## 4. Nonsense-Mediated mRNA Decay

Cells have evolved different mRNA surveillance mechanisms in order to evade a number of possible errors that can occur across the different steps of mRNA metabolism. Among these mechanisms, nonsense-mediated mRNA decay is one of the best characterized. Originally, NMD was identified as a post-transcriptional quality control mechanism responsible for the degradation of abnormal transcripts harboring PTCs, thus avoiding their translation into truncated proteins that could have either non-functional or dominant-negative effects [40]. However, in 2004, Mendell et al. [41] revealed that some normal mammalian transcripts are also NMD targets. Since then, those normal and fully functional NMD-targets have been under study to determine what genomic features are recognized by the NMD machinery. To date, NMD-inducing features include the presence of upstream open reading frames (uORFs), long 3′UTRs, or introns located more than 55 nucleotides (nts) downstream of the stop codon [42]. Nevertheless, some transcripts containing one or more of those features have been documented to evade NMD, such as the set of human mRNAs with long 3′UTRs identified by Singh et al. [43]. These results have raised the question of what other factors not necessarily embedded in the mRNA body could prevent or favor NMD, such as for example, 3′UTR-associated factors that stimulate or antagonize the recruitment of the NMD factor, UPF1 [43].

**Figure 1:**
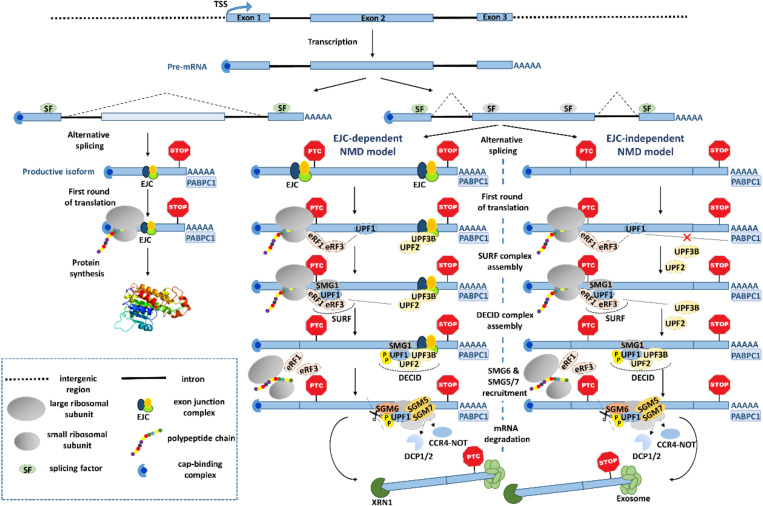
Representation of the exon junction complex (EJC)-dependent and EJC-independent nonsense-mediated RNA decay (NMD) pathway, targeting a transcript generated by alternative splicing (AS). Introns are represented as black lines and exons as blue boxes. A gene is transcribed into a pre-mRNA and AS gives rise to two different mRNA isoforms. The left one represents a productive isoform that encodes a functional protein, and the two isoforms on the right include a premature stop codon (PTC)-containing exon that triggers NMD. The EJC-dependent model: During the first round of translation, the ribosome encounters a stop codon located more than 50-55 nucleotides upstream of an exon-exon junction, and eRF1/3 interact with UPF1 and SMG1, resulting in the SURF complex. Then, UPF1 interacts with UPF2 at the EJC, which ends in the DECID complex formation and activation of UPF1 through phosphorylation by SMG1. The EJC-independent model: If the interaction between PABPC1 and eRF3 is inhibited when the ribosome reaches a PTC, eRF3 can interact with UPF1 originating the SURF complex. Then, UPF2 and UPF3B diffused in the cytoplasm interact with UPF1, triggering UPF1 phosphorylation by SMG1 and the DECID complex assembly. The next steps are common to both models: The active UPF1 leads to its helicase function, rearranging the transcript to allow the recruitment of SMG5, SMG6, and SMG7. SMG6 cleaves the mRNA near the PTC, which results in unprotected ends. Meanwhile, SMG5 and SMG7 bind as a heterodimer to recruit the DCP1 and DCP2 decapping enzymes and the CCR4-NOT deadenylation complex. These RNA modifications allow 5′-3′ and 3′-5′ degradation by XRN1 and the exosome, respectively. TSS: Transcription start site.

Two main models have been proposed for the NMD mechanism: The canonical one dependent on the exon junction complex (EJC), and the EJC-independent model (Figure 1). The EJC is a multiprotein complex that in most cases is deposited 20-24 nts upstream of the exon-exon junctions during splicing [44], and remains bound to the mRNA until the first round of translation. This complex allows the NMD machinery to distinguish between normal and premature termination codons. Indeed, if EJC(s) are located more than 50-55 nts downstream of the stop codon, the ribosome cannot displace them, rather, during translation termination at the stop codon, the termination complex can interact with the NMD machinery and trigger rapid decay [45]. More specifically, when the elongating ribosome encounters a stop codon located more than 50-55 nts upstream of one (or more) EJC(s), the eukaryotic release factors (eRF) 1 and 3, SMG1 kinase and the ATP-dependent helicase, UPF1, interact to form the SURF complex [46] which, in turn, interacts with the UPF2/UPF3B-containing EJC that results in the DECID complex. This leads to a conformational change in UPF1, allowing its phosphorylation by SMG1, and dissociation of eRF1 and eRF3 [47,48]. Active p-UPF1 leads to its helicase function, rearranging the transcript [49,50] to allow the recruitment of SMG5, SMG6, and SMG7. SMG6 is a conserved endonuclease that cleaves the mRNA in the vicinity of the nonsense codon, which results in unprotected ends leading to degradation [51]. Meanwhile, SMG5 and SMG7 bind as a heterodimer [52] to recruit decapping enzymes (DCP1 and DCP2) [53] and the CCR4-NOT deadenylation complex [54], which further results in XRN1-catalyzed 5′-3′ degradation and exosome-induced 3′-5′ decay, as a consequence of the absence of the 5′ cap and the 3′ poly(A) tail, respectively [55].

Interestingly, there is an increasing literature characterizing the EJC landscape, as for example, the study reported by Saulière et al. on the identification of transcriptome-wide binding sites of the EJC core component, eIF4A3, by deep sequencing after ultraviolet crosslinking and immunoprecipitation, the CLIP-seq method [56]. Although these authors observed a clear enrichment of eIF4A3 ~24 nts upstream of exon-exon junctions, they also localized this factor outside of canonical EJC positions. Indeed, according to the authors, only 50% of read peaks were consistent with canonical positions, and the vast majority of transcripts contained both canonical and non-canonical EJCs. These results resemble the ones published by Singh et al., reporting that the EJC peaks at non-canonical positions represent around 40% of total exonic peaks [57]. Moreover, such study shows Gene Ontology enrichment in canonical and non-canonical EJC occupancy on AS-NMD mediated gene expression regulation. The fact that EJC deposition does not always occur at canonical positions may partially explain differences in NMD efficiency and why some transcripts with PTCs located less than 50 nts upstream of the last exon-exon junction undergo splicing-dependent NMD [58,59].

Regarding the EJC-independent mechanism (Figure 1), it relies on the long physical distance between the translation termination reaction at the stop codon and the poly(A)-binding protein cytoplasmic 1 (PABPC1), which resides at the poly(A) tail. As PABPC1 and UPF1 both compete for the interaction with eRF3, if PABPC1 is distant from the stop codon, UPF1 can interact with the translation termination factor eRF3, signaling the stop codon as premature, and triggering NMD, even in the absence of EJC [60,61]. On the contrary, if PABPC1 is in close proximity to the termination complex, it prevents the UPF1-eRF3 interaction and inhibits NMD [43,62,63]. Interestingly, genome-wide studies measuring mRNA decay rates and/or gene expression levels of the whole transcriptome in UPF1-depleted cells have shown no correlation between the 3′UTR length and NMD activity [43,64,65]. One possible reason is that the physical distance that separates PABPC1 and the stop codon is not given just by the number of nucleotides, but by a spatial rearrangement of the 3′UTR, which can bring PABPC1 closer to the termination complex [66]. Additionally, when the PTC is proximal to the start codon (AUG-proximal PTC), PABPC1 can be brought into close proximity to the PTC via interactions with the cap-binding complex subunit eIF4G [67], promoted by a “closed-loop” configuration of the mRNA [68], resulting in NMD-inhibition. These data show that spatial 3D mRNP configuration may dictate the mRNA fate.

## 5. Alternative Splicing Coupled to NMD

**Figure 2:**
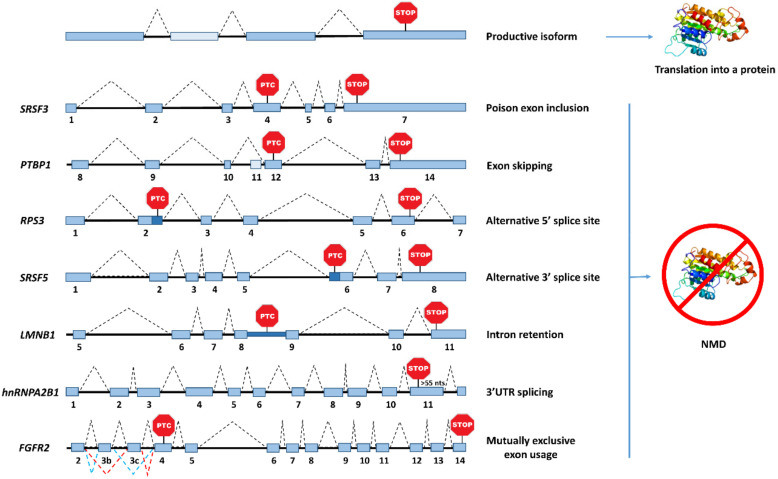
Alternative splicing (AS) patterns inducing inclusion of a premature termination codon (PTC). Introns are represented as black lines and exons as blue boxes. The first splicing pattern listed in the image creates a full-length productive isoform that encodes a functional protein. The other represented transcripts contain a PTC that commits the isoform to nonsense-mediated mRNA decay (NMD). Cassette exon events, such as poison exon inclusion, induce retention of a PTC-containing exon, as shown for *SRSF3* [78]. Exon skipping, another cassette exon event, may result in a frameshift leading to a PTC-positive isoform, as reported for *PTBP1* [79]. Usage of an alternative 5′ or 3′ splice site, as well as intron retention, can include a segment of RNA with an in-frame PTC, turning the transcript into an NMD target, as represented above for *RPS3*, *SRSF5*, and *LMNB1*, respectively [71,80,81,82]. Splicing in the 3′ untranslated region (UTR) of *hnRNPA2B1* can recruit an exon junction complex to a position located more than 55 nucleotides downstream of the stop codon, creating a premature context that triggers NMD [72]. The last example represents the inclusion of two exons in *FGFR2*, which are mutually exclusive, but if both exons are included or neither exon are in the splice variant, they introduce a frameshift that creates a PTC [83].

Alternative splicing is the main source of PTC-containing transcripts, and it is estimated that one third of all the AS events leads to the inclusion of an in-frame nonsense codon, thus committing such mRNAs to NMD [69]. During the last decade, genome-wide studies have unveiled a large amount of alternative splicing forms, which are actually targets of NMD. These data suggested that the entire pool of unproductive transcripts could not simply represent biological noise, but at least partially, a mechanism of gene expression regulation. Alternative splicing patterns, such as cassette exons, which include or exclude an exon from the transcript, alternative 5′/3′ splice sites or intron retention in the 3′UTR, are frequent splicing events leading to PTC-containing isoforms, that in turn trigger NMD (Figure 2) [70,71]. Particularly, a poison cassette exon induces the retention of a PTC-containing exon, while an exon skipping event, may result in a frameshift that induces a downstream PTC. On the other hand, alternative 5′/3′ splice sites and intron retention, can include a sequence in the mRNA with an in-frame PTC (Figure 2). Alternative splicing in the 3′UTR can also induce NMD, due to the presence of EJC(s) located >50–55 nts downstream of the normal stop codon [72]. Therefore, AS uses several stratagems to commit an mRNA to degradation by NMD. Indeed, published data supports the idea that the decay of the non-functional RNA isoforms is central for the cell, as the encoded non-functional protein would be deleterious. To understand the importance of decay of an AS RNA by NMD, it would be interesting to investigate the biological consequences of subtly altering an AS RNA encoding a non-functional protein so that it would encode precisely the same non-functional protein but the RNA encoding it would no longer be degraded by NMD. To our knowledge, this experiment has not yet been performed, which constitutes a major hole in the field.

This coordinated action between alternative splicing and NMD, known as AS-NMD, was proposed as a post-transcriptional regulatory process of the cell, also called “Regulated Unproductive Splicing and Translation” (RUST), to achieve the proper expression level of a given protein by degrading some fraction of the already-transcribed mRNA [69,73]. Here, splicing factors play a key role shifting the balance of splice sites towards productive transcripts or NMD-targeted isoforms. In fact, a very well-known example of AS-NMD event is the autoregulatory negative feedback loop observed for many RBPs, especially splicing factors, and some other core spliceosomal and ribosomal proteins. Accordingly, such RBPs recognize and bind their own pre-mRNAs, inducing non-productive PTC-containing AS RNAs, which are degraded by NMD in order to autoregulate their protein steady-state levels. Moreover, it is well known that mutations in the SR and hnRNP families of splicing factors abolish this negative feedback loop, while their overexpression increases NMD-sensitive isoforms [72,74,75,76,77]. There are also cross regulatory AS-NMD events between splicing factors, as we discuss in the following sections. This clearly indicates that AS coupled to NMD is an important post-transcriptional regulatory step of gene expression for RBPs, especially for the different families of splicing factors. However, it is still necessary to identify which unproductive alternative splicing events represent AS-NMD-mediated gene expression regulation, or mis-spliced RNAs that would rather be degraded by NMD.

Interestingly, AS-NMD operates in orthologue genes of such phylogenetically distant organisms as mammals, plants, or yeast [73]. One of the first and best characterized examples of an evolutionarily conserved AS-NMD event is the regulation of the human *PTBP1* gene, whose negative feedback loop inducing an NMD-isoform has also been observed in other species, such as *Xenopus laevis* and *Fugu rubripes* [79,84]. Another important example is the ultra-conserved NMD-inducing exons detected in SR and hnRNP protein families and core spliceosomal members, indicating that this process is highly conserved across different species [71,85]. Moreover, AS-NMD regulation plays an important role in cell differentiation and tissue-specific gene expression, with deleterious outcomes in the case of misregulation [82,86,87,88]. As discussed in the following sections, altered AS-NMD regulatory events are linked to several human diseases, including cancer. Such biological consequences together with the high degree of conservation in crucial gene expression regulatory factors evidences AS-NMD as a functionally important pathway in the cell. However, whether its function is limited to controlling mRNA steady-state levels of certain RBPs or expanding upon this action remains unclear. Clarification is also needed for its redundancy with respect to other post-transcriptional gene expression regulation mechanisms, and in which scenarios its action is triggered, other than protein abundancy.

## 6. Splicing Factors are Autoregulated by AS-NMD

Among the SR protein family, SRSF2, also known as SC-35, constitutes the first example of a SR protein capable of regulating its own expression by AS-NMD [74]. Sureau et al. reported that SRSF2 overexpression in HeLa cells results in lower *SRSF2* mRNA levels, as well as different splicing patterns. Particularly, this SR protein targets its own pre-mRNA to induce both, inclusion of a poison cassette exon and intron retention in the 3′UTR [74], the latter forcing the normal termination codon to be recognized as premature, thus being subjected to NMD. This negative autoregulation has also been documented in other human classical SR proteins, such as SRSF1, SRSF3, SRSF4, SRSF5, or SRSF7 (Table 1), due to different splicing events [71,75,77,78,80,81,89,90,91]. For example, in the case of SRSF5, the usage of the proximal 3′ splice site in the exon 6 includes an in-frame stop codon that induces NMD [81]. Interestingly, this type of autoregulation can involve multiple layers, as observed for SRSF1, which negatively controls its own expression not only by AS-NMD, but also by other post-transcriptional mechanisms, such as nuclear retention of some alternative spliced isoforms, that will end in non-protein production [75].

Regarding the hnRNP family of splicing factors, they are also autoregulated by AS-NMD, but following an opposite strategy relative to what is observed for SR proteins. Given that most of these factors behave as splicing repressors, high levels of hnRNPs promote exon skipping, inducing a frameshift that gives rise to PTC-containing isoforms, which, in turn, results in increased mRNA turnover [89]. Wollerton et al. reported the first case of this negative feedback in the polypyrimidine tract binding protein 1 (PTBP1), also known as hnRNP I [79] (Table 1). High PTBP1 protein levels induce alternative skipping of exon 11 in its own mRNA, introducing a downstream in-frame PTC that triggers NMD, hence reducing the PTBP1 protein levels. High-throughput methods have contributed deeply to the current knowledge in this field. For instance, coupling depletion of key NMD factors to mass spectrometry allowed the identification of an autoregulatory feedback mechanism controlling homeostasis of the hnRNPA2B1 protein. McGlincy et al. found that the UPF1 knockdown led to the detection of an NMD-sensitive isoform with a 3′UTR intron spliced out, creating an exon-exon junction that places the normal termination codon in a premature context [72]. Then, they confirmed that overexpression of hnRNPA2 reduces *hnRNPA2* and *hnRNPB1* mRNA levels and increases NMD-sensitive isoforms containing EJCs downstream of the stop codon. Nevertheless, there are some documented exceptions of hnRNPs using a mechanism of action similar to that used by SR proteins. An example is hnRNP L, which functions as a splicing enhancer that promotes the inclusion of a short exon embedded in intron 6, containing a PTC [76] (Table 1).

In addition to SR and hnRNP proteins, other RBPs can also catalyze the splicing of nonproductive isoforms. The snRNP SNRPB (also known as SmB/B’) constitutes a good example of a core spliceosomal component controlling its own protein homeostasis through the inclusion of a PTC-positive alternative exon flanked by highly conserved intronic sequences [92,93] (Table 1). While SNRPB knockdown in HeLa cells leads to increased skipping of an NMD-inducing exon, its overexpression results in a higher fraction of transcripts targeted by NMD [93]. Moreover, ribosomal proteins undergo AS-NMD mediated regulation, and represent pioneer studies in the understanding of this mechanism in *Caenorhabditis elegans* [94]. Cuccurese et al. reported for the first time an autoregulatory negative feedback loop for the human RPL3 ribosomal protein (Table 1). The authors observed that overexpression of RPL3 leads to the 3′ splice site usage in intron 3, resulting in a partial intron retention [95]. The inclusion of the alternatively spliced region, consequently, generates an in-frame PTC located > 55 nts upstream of the last exon-exon junction, thus committing the mRNA to degradation.

## 7. Cross-Regulation between Splicing Factors

As discussed above, autoregulation by AS-NMD is a common mechanism in several human RBPs to maintain proper protein expression. However, far from being an isolated process in the cell, RBPs interact with other proteins to fine-tune their alternative splicing choices and ultimately lead to a certain fraction of productive isoforms.

An increasing number of studies has been published during the last decade about the interplay between splicing factors in order to balance the ratio of productive isoforms, constituting an important layer of post-transcriptional regulation. This is commonly found in paralog proteins, probably due to ultra-conserved regulatory *cis-*elements that assist the recognition of closely related proteins [71]. That is the case of the hnRNP protein PTBP1, which cross-regulates the expression of its neural paralog PTBP2, also known as nPTB [98] (Table 2). Spellman et al. detected increased levels of PTBP2 protein upon PTBP1 knockdown in HeLa cells. This observation is explained since PTBP1 regulates PTBP2 splicing, inducing exon 10 skipping, which originates a transcript with a downstream PTC that triggers rapid mRNA decay. In addition to this AS-NMD-mediated regulation, there is a compensation mechanism between these two factors due to the redundancy they show in terms of targets, as suggested by the fact that the PTBP1 knockdown has very little effect in the HeLa cells proteome [98]. Another good example is the cross-regulatory relationship between hnRNP L and hnRNP LL (Table 2), both operating in alternative splicing events [99,100]. Rossbach et al. documented that hnRNP L depletion in HeLa cells induces hnRNP LL up-regulation of both mRNA and protein levels by AS-NMD. They showed that *hnRNP LL* contains a potential poison exon responsive to NMD, whose inclusion is promoted by hnRNP L [76]. In addition, reciprocal cross-regulation, meaning two proteins controlling the expression of one another by AS-NMD, has been shown recently for hnRNP D and its paralog hnRNP DL [101]. Both proteins regulate their own expression by an autoregulatory negative feedback loop that induces alternative splicing of cassette exons in the 3′UTR. Exon 8 inclusion in *hnRNP DL* mRNA produces two exon junctions, the second one located >55 nts downstream of the normal termination codon, which triggers NMD. Similarly, hnRNP D targets its own pre-mRNA promoting the inclusion of exon 9, which results in lower protein levels [101]. Interestingly, the production of spliced forms with EJCs downstream of the stop codon can also be coordinated between hnRNP D and hnRNP DL (Table 2), so that each of these splicing factors regulates its own transcripts and those of the other factor.

The SR family of splicing factors has also developed this crosstalk regulation to alternatively splice unproductive isoforms. CLIP-seq allows mapping of protein-RNA binding sites, being a potent high-throughput approach to detect new cross-regulatory feedbacks between splicing factors. Änkö et al. applied this method to the SRSF3 protein, and besides showing how it modulates its own alternative splicing, which was already documented in previous studies [78,102], they reveal that SRSF3 binds to poison cassette exons of other SR proteins, such as SRSF5 and SRSF7, triggering their decay [90] (Table 2). Using the same approach, Jangi et al. identified hundreds of AS-NMD splicing events regulated by the RNA-binding protein, Rbfox2, in mouse embryonic stem cells (Table 2). They found that many of the targets cross-regulated by Rbfox2 were RBPs capable of autoregulation by AS-NMD, creating a complex network where Rbfox2 fine-tunes their mRNA levels [103]. They experimentally demonstrated that this master regulator enhances or represses the pool of NMD isoforms of these RBPs depending on the target in a context-dependent manner. Altogether, these data show that auto- and cross-regulatory AS-NMD events constitute entire networks that seem to tightly control the protein production of several splicing factors and, therefore, the splicing pattern of many other transcripts, having an overall impact in the cellular proteome.

## 8. AS-NMD in Cell Differentiation and Tissue-Specific Gene Expression Regulation

The complexity of cell differentiation during embryonic development or other physiological processes such as hematopoiesis have been extensively characterized as a spatiotemporal-dependent process where gene expression regulation is mainly orchestrated by *cis*-regulatory DNA sequences, known as enhancers and promoters, that allow the recruitment of a variety of transcription factors [105]. However, the contribution of AS-NMD in fine-tuning overall gene expression also takes part in cell differentiation and tissue-specific gene expression regulation. The first case reporting the involvement of this regulatory pathway in tissue specificity was for the *MID1* gene, which encodes a protein that plays a role in protein recycling by ubiquitin tagging. Winter et al. observed several splice variants for *MID1* in a tissue-specific manner, and also at different developmental stages comparing expression patterns in adult- and fetal-derived cells [86]. Those AS RNAs are mainly the product of three types of event, two of them creating novel exons containing in-frame start or stop codons that give rise to N- and C-terminally truncated proteins, respectively, and a third class of transcripts including a premature stop codon that commits the isoform to NMD. Interestingly, distinct transcript variants were detected in different cell types, such as fibroblasts, liver, or brain cells [86]. Another example that supports the importance of AS-NMD in cell differentiation was described by Wong et al. [82]. These authors reported intron retention coupled to NMD as a crucial mechanism that regulates granulocyte differentiation in mouse bone marrow. The authors used parallel mRNA sequencing and mass spectrometry on cells isolated at different stages of granulopoiesis, which allowed them to identify intron retention as a programmed splicing event committing important mRNAs in myeloid differentiation to NMD [82].

Transcript regulation of the *postsynaptic density protein 95* (*PSD-95*) gene (also known as *DLG4*) is another well characterized example of a gene subject to AS-NMD regulation, in this case leading to neural-specific expression [87,88]. Two hnRNPs, PTBP1 and PTBP2 regulate *PSD-95* alternative splicing, inducing skipping of exon 18, which causes a shift in the reading frame that originates a PTC. Therefore, exon 18-depleted transcripts are targeted by NMD and, consequently, there is no protein synthesis. Conversely, the low expression of PTBP1 and PTBP2 in neurons derepresses splicing inclusion of exon 18, allowing PSD-95 protein expression. This represents a very important step in mammalian neural development, since *PSD-95* mRNA is mostly degraded in early embryonic brains and translated into protein during neuronal maturation [87]. Impairment of this regulatory mechanism results in severe deleterious outcomes, such as an inappropriate development of glutamatergic synapses.

These data indicate that AS-NMD has a relevant physiological role in determining tissue-specific gene expression and cell differentiation, by governing which splice variants are produced and limiting protein production to certain cell types.

## 9. AS-NMD Dysfunction and Associated Human Diseases

Deregulation of AS-NMD mediated gene expression represents the cause of many cancer types, as well as some neurological and cardiovascular disorders.

### 9.1. Misregulation of AS-NMD and Cancer

Many myelodysplastic syndromes and solid tumors are frequently caused by oncogenic mutations in splicing factors, which originate genome-wide splicing abnormalities affecting the expression of cancer-related genes [106,107,108,109,110,111]. Different biological consequences from these mutations have been documented, turning an RNA splicing factor into an oncoprotein or a tumor suppressor, depending on the context. A well-characterized example is SRSF2, a splicing factor with a stimulatory effect on NMD [112,113], which is commonly mutated in Pro95 in patients affected by acute myeloid leukemia (AML) [111,114,115,116]. This mutation changes the RNA-binding affinity of SRSF2, mis-regulating the splicing pattern of many of its targets [116,117]. Interestingly, Rahman et al. revealed that the mutated SRSF2 over-promotes mRNA decay by NMD, since its binding to a given target increases EJC recruitment, which provides a stronger association with the NMD machinery [111]. One of the targets mis-spliced by SRSF2^Mut^ is *EZH2* [111,116], a key enzymatic subunit of the methyltransferase Polycomb repressive complex 2 (PRC2). This gene is frequently dysregulated in several tumors [118,119,120], displaying an oncogenic or tumor suppressor activity, depending on the cancer type [121]. SRSF2^Mut^ binds to a C-rich ESE, driving the inclusion of a *EZH2* poison exon that induces NMD and reduces EZH2 protein levels [111,116]. Moreover, in agreement with these results, previous studies reported that the *EZH2* loss-of-function mutations and SRSF2^Mut^ occur in the same spectrum of malignant myeloid disorders, where EZH2 seems to behave as a tumor suppressor [122,123,124].

Splicing factors regulating AS-NMD can also function as oncogenes, as described for SRSF1 [125,126] and SRSF3 [77,127]. SRSF1 controls alternative splicing of the proto-oncogene *MST1R* (also known as *Ron*) [125], whose active isoform accumulates in different cancer types and translates into a tyrosine kinase receptor that increases cell mobility, invasion, and resistance to apoptosis-induced death [125,128,129,130,131,132]. This MST1R productive isoform is prompted by SRSF1, inducing skipping of exon 11, and ultimately inducing the epithelial–mesenchymal transition (EMT) [125]. Interestingly, upstream in this pathway, AS-NMD regulates the fraction of the SRSF1 productive isoform by the action of another splicing factor, KHDRBS1 [104]. Under physiological conditions, SRSF1 retains an intron that commits the transcript to NMD. Nevertheless, during the EMT program, KHDRBS1 increases the *SRSF1* transcript stability, thus positively modulating its protein production [104], which, in turn, increases the expression of the oncoprotein MST1R.

Regarding the splicing factor SRSF3, increased levels are detected in several cancers [133,134]. Interestingly Guo et al. found that cross-regulation between hnRNPs and this SR protein is the mechanism responsible for its overexpression [77]. As documented for other SR proteins, SRSF3 autoregulates its gene expression by promoting the splicing of exon 4, which contains an in-frame PTC [77,78]. However, in cancer cells, the hnRNP splicing factors PTBP1 and PTBP2, are able to impair this negative feedback mechanism by binding to an ESS in exon 4 of *SRSF3*, inhibiting its inclusion and promoting SRSF3 upregulation. In order to confirm that high SRSF3 levels are required for the tumorigenic phenotype, Guo et al. depleted SRSF3 in cells of oral squamous carcinoma and observed a significant inhibition of cell growth [77]. These data highlight the relevance of cross-regulatory AS-NMD pathways for normal cell function, and how its mis-regulation can result in carcinogenesis.

Worth mentioning, AS-NMD seems to be altered under hypoxia, a stressful state experienced by most malignant tumors [135]. This was observed for the *Cysteine-rich angiogenic inducer 61* (*CYR61*) gene, which is regulated by AS-NMD and encodes a matricellular protein that favors distinct hallmarks of cancer, such as cell proliferation, migration, survival, or angiogenesis in different tumors [136,137,138,139,140]. In physiological conditions, this gene is under the posttranscriptional control of AS-NMD, which induces the retention of intron 3, leading to an intron-retaining phenotype that yields an NMD-sensitive isoform [141]. However, under hypoxia, this AS-NMD pathway is altered, inducing skipping of intron 3, and hence, promoting the formation of a productive isoform, which is translated into an active protein with proangiogenic properties [141]. Hypoxia also influences splicing patterns of some splicing factors, such as YT521, which targets cancer-related genes and has been associated with a tumor suppressor activity [96,142]. Expression of the *YT521* gene can be autoregulated by AS-NMD through skipping of exons 8 and 9, creating an NMD-sensitive isoform by the acquisition of a downstream PTC. Interestingly, under hypoxic conditions, this gene experiences a switch in its splicing pattern, that results in non-productive isoforms, which are coupled to NMD [96]. Consequently, the reduction of protein levels impacts the splicing isoforms of YT521 cancer-related targets, such as *BRCA2* and *PGR*. Nevertheless, further analyses are needed to assess the impact of YT521 knockdown on key hallmarks of cancer.

### 9.2. Other Disorders Associated with AS-NMD Misregulation

Aberrant alternative splicing resulting in low expression of productive isoforms due to NMD induction can also be the cause of a neurodegenerative disorder, as for example, amyotrophic lateral sclerosis (ALS). This disease is caused by abnormal protein aggregates in the cytoplasm of motor neurons. Such aggregates have a high associated toxicity and are commonly originated by FUS and TDP-43, both RNA- and DNA-binding proteins with numerous functions, including alternative splicing [143,144]. The pathology associated with ALS commonly arises from high levels of mis-spliced *FUS* and *TDP-43* mRNAs [145,146], which presumably could overload the NMD machinery due to the elevated rate of aberrant transcripts and result in overproduction of truncated proteins, as suggested by Jaffrey and Wilkinson [147]. Therefore, this widespread production of truncated proteins might induce neural toxicity and promote ALS. Indeed, there are studies reporting how an increased activity of the NMD pathway can reduce the ALS-associated toxicity [148,149]. In addition, FUS is able to autoregulate its protein abundance by AS-NMD through the repression of exon 7 splicing, and mutant variants of this ALS-related splicing factor have been directly correlated with aberrant autoregulation [97]. This suggests that the impaired AS-NMD-mediated regulation of FUS can contribute to ALS development, explaining its characteristic cytoplasmic aggregates found in patients.

Most of the disease-related mutations found in spliceosomal components, splicing factors, or splice sites have been associated with cancer and some neurological disorders. However, defects in the regulation of productive isoforms by AS-NMD have also been reported in other diseases, such as myotonic dystrophy (DM), the most frequent autosomal muscular dystrophy in adults [150]. A trinucleotide (CTG) repeat expansion in the 3′UTR of the *myotonic dystrophy protein kinase* (*DMPK*) gene causes myotonic dystrophy and disrupts the normal function of the CELF1 splicing factor. However, the mechanism by which such trinucleotide expansion affects the function of CELF1 remains unknown. Some authors explain that this disruption is due to indirect effects, for example, hyperphosphorylation of CELF1 by the protein kinase PKC, which stabilizes the protein [151], or reduces the levels of miR-23a/b, a miRNA that suppresses CELF1 translation [152,153]. Therefore, this repeat expansion results in a gain of CELF1 activity that contributes to the DM pathogenesis [154,155]. One of the main targets affected by the dysregulated CELF1 is a muscle-specific chloride channel (*CLCN1*). The splicing pattern of this gene has been deeply characterized by Nakamura et al. who revealed that *CLCN1* expression is driven via AS by CELF1 among other splicing factors and presents a splice variant carrying a PTC [156]. The CELF1 gain of function reported in DM induces a switch in the *CLCN1* splicing pattern towards a higher fraction of AS RNAs containing a PTC, which deeply downregulates its protein expression [157,158]. Indeed, rescue experiments restoring the full-length reading frame of *CLCN1* abolished the myotonic pathology in mice [159]. These results suggest that the AS-NMD regulation could explain the molecular mechanism, which in some cases drives muscular dystrophy.

Another disease related to the disruption of a splicing factor is dilated cardiomyopathy (DCM), a heart disease caused by the loss of SRSF2 [160]. Ding et al. induced ablation of this splicing factor in the heart using a transgenic mouse and observed the DCM phenotype 3-5 weeks after birth. Then, they searched for changes in gene expression across the transcriptome and detected that the *cardiac specific ryanodine receptor 2* (*RyR2*) was downregulated, showing that such dysregulation leads to a specific excitation-contraction defect on isolated cardiomyocytes [160]. Authors suggest that the mechanisms behind the lower RyR2 protein levels are a direct consequence of SRSF2 depletion, which promote the formation of a mis-spliced *RyR2* isoform targeted by NMD.

## 10. Conclusions

In the past decade, the RNA biology field has experienced huge progress, especially with respect to RNA splicing. Cutting-edge technologies, such as next-generation sequencing, and its wide application breadth have significantly contributed to the knowledge in this area. The increasingly popular use of this technology by the scientific community has provided valuable transcriptomic data regarding the existing AS RNAs for a particular gene, and in which splicing patterns occur for a given scenario. This has led to many and varied examples of AS-NMD mediated regulation, which brings us closer to deciphering the functional significance of this biological process. So far, it is well known that AS-NMD is able to fine-tune the levels of many RNA-binding proteins by balancing the ratio between productive and non-productive mRNA isoforms. Given that many of these RBPs are splicing factors, dysregulation of this regulatory pathway can have widespread effects over the transcriptome, affecting the splicing patterns of downstream genes, compromising the normal function of the corresponding physiological processes, and leading to the appearance of multiple diseases. Therefore, the knowledge of how the AS-NMD pathway operates and in which situations, provides data of value for the development of therapeutic approaches that could alleviate the protein shortages, which frequently cause or exacerbate a given disorder, as well as provide prognostic biomarkers, ensuring proper treatment at the right moment.

## Figures and Tables

**Table 1:** Human RNA-binding proteins autoregulated by alternative splicing coupled to nonsense-mediated mRNA decay.

AS-NMD Autoregulated Proteins	Gene Name	Splicing Pattern	References
SR proteins	*SRSF1*	3′UTR intron retention	Ni et al. (2007) [89]; Sun et al. (2010) [75]
	*SRSF2*	Poison cassette exon/3′UTR intron retention	Sureau et al. (2001) [74]
	*SRSF3*	Poison cassette exon	Jumma and Nielsen (1997) [78]
	*SRSF4*	Poison cassette exon	Lareau et al. (2007) [71]; Änkö et al. (2012) [90]
	*SRSF5*	Alternative 3′ splice site	Lareau et al. (2007) [71]; Yang et al. (2018) [81]
	*SRSF7*	Poison cassette exon	Laureau et al. (2007) [71]; Königs et al. (2020) [91]
*hnRNP proteins*	*PTBP1*	Exon skipping	Wollerton et al. (2004) [79]
	*hnRNPA2B1*	3′UTR splicing	McGlincy et al. (2010) [72]
	*hnRNP L*	Poison cassette exon	Rossbach et al. (2009) [76]
Spliceosome components	*SNRPB*	Poison cassette exon	Saltzman et al. (2008) [92]; Saltzman et al. (2011) [93]
Ribosomal proteins	*RPL3*	Alternative 3′ splice site	Cuccurese et al. (2005) [95]
Others	*YT521*	Exon skipping	Hirschfeld et al. (2013) [96]
	*FUS*	Exon skipping	Zhou et al. (2013) [97]
	*SFPQ*	3′UTR splicing	Pervouchine et al. (2019) [80]
	*RPS3*	Alternative 5′ splice site	

**Table 2:** Human mRNA binding proteins regulated by alternative splicing coupled to nonsense-mediated mRNA decay cross-regulatory events.

mRNA-Binding Protein	Target	Splicing Event	References
PTBP1	PTBP2	Exon skipping	Spellman et al. (2007) [98]
PTBP1/PTBP2	PSD-95	Exon skipping	Zheng et al. (2012) [87]
hnRNP L	hnRNP LL	Poison cassette exon	Rossbach et al. (2009) [76]
hnRNP D	hnRNP DL	3′UTR splicing	Kemmerer et al. (2018) [101]
hnRNP DL	hnRNP D		
SRSF3	SRSF5/7	Poison cassett exon	Änkö et al. (2012) [90]
KHDRBS1	SRSF1	3′UTR splicing	Valacca et al. (2010) [104]
Rbfox2	>70 RBPs	Cassette exon	Jangi et al. (2014) [103]

## Abbreviations

ABCA7	ATP-binding cassette subfamily A member 7
AD	Alzheimer’s diseases
ALS	Amyotrophic lateral sclerosis
AML	Acute myeloid leukemia
AS	Alternative splicing
AS-NMD	Alternative splicing coupled to NMD
CLIP-seq	Cross-linking immunoprecipitation sequencing
CYR61	Cysteine-rich angiogenic inducer 61
DCM	Dilated cardiomyopathy
DM	Myotonic dystrophy
DMPK	Myotonic dystrophy protein kinase
EJC	Exon junction complex
EMT	Epithelial-mesenchymal transition
eRF	Eukaryotic release factor
ESE	Exonic splicing enhancer
ESS	Exonic splicing silencer
hnRNP	Heterogeneous nuclear ribonucleoprotein
ISE	Intronic splicing enhancer
ISS	Intronic splicing silencer
mRNP	Messenger ribonucleoprotein particle
NMD	Nonsense-mediated mRNA decay
nt	Nucleotide
PABPC1	Poly(A)-binding cytoplasmic protein 1
PRC2	Polycomb repressive complex 2
PTB	Polypyrimidine tract-binding protein
PTBP1	Polypyrimidine tract-binding protein 1
PTC	Premature termination codon
RBM20	RNA binding motif protein 20
RBP	RNA-binding protein
RUST	Regulated Unproductive Splicing and Translation
RyR2	Cardiac specific ryanodine receptor 2
SMA	Spinal muscular atrophy
SMN1	Survival motor neuron 1
snRNP	Small nuclear ribonucleoprotein
SR	Serine arginine-rich
SRSF	Serine arginine-rich splicing factor
uORF	Upstream open reading frame
UTR	Untranslated region

## References

[1] Burge C.S., Tuschl T., Sharp P.A. (1999). Splicing of Precursors to mRNAs by the Spliceosomes. The RNA World.

[2] Jurica M.S., Moore M.J. (2003). Pre-mRNA splicing: Awash in a sea of proteins. Mol. Cell.

[3] Maquat L.E., Carmichael G.G. (2001). Quality control of mRNA function. Cell.

[4] Bentley D.L. (2005). Rules of engagement: Co-transcriptional recruitment of pre-mRNA processing factors. Curr. Opin. Cell Biol..

[5] Will C.L., Lührmann R. (2011). Spliceosome structure and function. Cold Spring Harb. Perspect. Biol..

[6] Black D.L. (2003). Mechanisms of alternative pre-messenger RNA splicing. Annu. Rev. Biochem..

[7] Martinez-Contreras R., Cloutier P., Shkreta L., Fisette J.F., Revil T., Chabot B. (2007). hnRNP proteins and splicing control. Adv. Exp. Med. Biol..

[8] Huelga S.C., Vu A.Q., Arnold J.D., Liang T.D., Liu P.P., Yan B.Y., Donohue J.P., Shiue L., Hoon S., Brenner S. (2012). Integrative genome-wide analysis reveals cooperative regulation of alternative splicing by hnRNP proteins. Cell Rep..

[9] Geuens T., Bouhy D., Timmerman V. (2016). The hnRNP family: Insights into their role in health and disease. Hum. Genet..

[10] Jeong S. (2017). SR proteins: Binders, regulators, and connectors of RNA. Mol. Cells.

[11] Zhou Z., Fu X.D. (2013). Regulation of splicing by SR proteins and SR protein-specific kinases. Chromosoma.

[12] Mayeda A., Helfman D.M., Krainer A.R. (1993). Modulation of exon skipping and inclusion by heterogeneous nuclear ribonucleoprotein A1 and pre-mRNA splicing factor SF2/ASF. Mol. Cell. Biol..

[13] Caputi M., Mayeda A., Krainer A.R., Zahler A.M. (1999). hnRNP A/B proteins are required for inhibition of HIV-1 pre-mRNA splicing. EMBO J..

[14] Valcárcel J., Gebauer F. (1997). Post-transcriptional regulation: The dawn of PTB. Curr. Biol..

[15] Spellman R., Rideau A., Matlin A., Gooding C., Robinson F., McGlincy N., Grellscheid S.N., Southby J., Wollerton M., Smith C.W.J. (2005). Regulation of alternative splicing by PTB and associated factors. Biochem. Soc. Trans..

[16] Schaub M.C., Lopez S.R., Caputi M. (2007). Members of the heterogeneous nuclear ribonucleoprotein H family activate splicing of an HIV-1 splicing substrate by promoting formation of ATP-dependent spliceosomal complexes. J. Biol. Chem..

[17] Wang E., Mueller W.F., Hertel K.J., Cambi F. (2011). G run-mediated recognition of proteolipid protein and DM20 5′ splice sites by U1 small nuclear RNA is regulated by context and proximity to the splice site. J. Biol. Chem..

[18] Shen M., Mattox W. (2012). Activation and repression functions of an SR splicing regulator depend on exonic versus intronic-binding position. Nucleic Acids Res..

[19] Chandler D.S., Qi J., Mattox W. (2003). Direct repression of splicing by transformer-2. Mol. Cell. Biol..

[20] Kanopka A., Muhlemann O., Akusjarvi G. (1996). Inhibition by SR proteins splicing of a regulated adenovirus pre-mRNA. Nature.

[21] Shin C., Manley J.L. (2002). The SR protein SRp38 represses splicing in M phase cells. Cell.

[22] Pan Q., Shai O., Lee L.J., Frey B.J., Blencowe B.J. (2008). Deep surveying of alternative splicing complexity in the human transcriptome by high-throughput sequencing. Nat. Genet..

[23] Wang E.T., Sandberg R., Luo S., Khrebtukova I., Zhang L., Mayr C., Kingsmore S.F., Schroth G.P., Burge C.B. (2008). Alternative isoform regulation in human tissue transcriptomes. Nature.

[24] Nilsen T.W., Graveley B.R. (2010). Expansion of the eukaryotic proteome by alternative splicing. Nature.

[25] Hughes T.A. (2006). Regulation of gene expression by alternative untranslated regions. Trends Genet..

[26] Pritsker M., Doniger T.T., Kramer L.C., Westcot S.E., Lemischka I.R. (2005). Diversification of stem cell molecular repertoire by alternative splicing. Proc. Natl. Acad. Sci. USA.

[27] Wu J.Q., Habegger L., Noisa P., Szekely A., Qiu C., Hutchison S., Raha D., Egholm M., Lin H., Weissman S. (2010). Dynamic transcriptomes during neural differentiation of human embryonic stem cells revealed by short, long, and paired-end sequencing. Proc. Natl. Acad. Sci. USA.

[28] Salomonis N., Schlieve C.R., Pereira L., Wahlquist C., Colas A., Zambon A.C., Vranizan K., Spindler M.J., Pico A.R., Cline M.S. (2010). Alternative splicing regulates mouse embryonic stem cell pluripotency and differentiation. Proc. Natl. Acad. Sci. USA.

[29] Han H., Irimia M., Ross P.J., Sung H.K., Alipanahi B., David L., Golipour A., Gabut M., Michael I.P., Nachman E.N. (2013). MBNL proteins repress ES-cell-specific alternative splicing and reprogramming. Nature.

[30] Su C.H., Dhananjaya D., Tarn W.Y. (2018). Alternative splicing in neurogenesis and brain development. Front. Mol. Biosci..

[31] Paronetto M.P., Passacantilli I., Sette C. (2016). Alternative splicing and cell survival: From tissue homeostasis to disease. Cell Death Differ..

[32] Blue R.E., Curry E.G., Engels N.M., Lee E.Y., Giudice J. (2018). How alternative splicing affects membrane-trafficking dynamics. J. Cell Sci..

[33] Schaub A., Glasmacher E. (2017). Splicing in immune cells-mechanistic insights and emerging topics. Int. Immunol..

[34] Ergun A., Doran G., Costello J.C., Paik H.H., Collins J.J., Mathis D., Benoist C. (2013). Differential splicing across immune system lineages. Proc. Natl. Acad. Sci. USA.

[35] Bechara E.G., Sebestyén E., Bernardis I., Eyras E., Valcárcel J. (2013). RBM5, 6, and 10 differentially regulate NUMB alternative splicing to control cancer cell proliferation. Mol. Cell.

[36] Choudhury R., Roy S.G., Tsai Y.S., Tripathy A., Graves L.M., Wang Z. (2014). The splicing activator DAZAP1 integrates splicing control into MEK/Erk-regulated cell proliferation and migration. Nat. Commun..

[37] Kim E., Magen A., Ast G. (2007). Different levels of alternative splicing among eukaryotes. Nucleic Acids Res..

[38] Elkon R., Ugalde A.P., Agami R. (2013). Alternative cleavage and polyadenylation: Extent, regulation and function. Nat. Rev. Genet..

[39] Tian B., Manley J.L. (2016). Alternative polyadenylation of mRNA precursors. Nat. Rev. Mol. Cell Biol..

[40] Culbertson M.R., Leeds P.F. (2003). Looking at mRNA decay pathways through the window of molecular evolution. Curr. Opin. Genet. Dev..

[41] Mendell J.T., Sharifi N.A., Meyers J.L., Martinez-Murillo F., Dietz H.C. (2004). Nonsense surveillance regulates expression of diverse classes of mammalian transcripts and mutes genomic noise. Nat. Genet..

[42] Hug N., Longman D., Cáceres J.F. (2015). Mechanism and regulation of the nonsense-mediated decay pathway. Nucleic Acids Res..

[43] Singh G., Rebbapragada I., Lykke-Andersen J. (2008). A competition between stimulators and antagonists of Upf complex recruitment governs human nonsense-mediated mRNA decay. PLoS Biol..

[44] Le Hir H., Izaurralde E., Maquat L.E., Moore M.J. (2000). The spliceosome deposits multiple proteins 20–24 nucleotides upstream of mRNA exon-exon junctions. EMBO J..

[45] Le Hir H., Gatfield D., Izaurralde E., Moore M.J. (2001). The exon-exon junction complex provides a binding platform for factors involved in mRNA export and nonsense-mediated mRNA decay. EMBO J..

[46] Kashima I., Yamashita A., Izumi N., Kataoka N., Morishita R., Hoshino S., Ohno M., Dreyfuss G., Ohno S. (2006). Binding of a novel SMG-1-Upf1-eRF1-eRF3 complex (SURF) to the exon junction complex triggers Upf1 phosphorylation and nonsense-mediated mRNA decay. Genes Dev..

[47] Yamashita A., Ohnishi T., Kashima I., Taya Y., Ohno S. (2001). Human SMG-1, a novel phosphatidylinositol 3-kinase-related protein kinase, associates with components of the mRNA surveillance complex and is involved in the regulation of nonsense-mediated mRNA decay. Genes Dev..

[48] Melero R., Uchiyama A., Castaño R., Kataoka N., Kurosawa H., Ohno S., Yamashita A., Llorca O. (2014). Structures of SMG1-UPFs complexes: SMG1 contributes to regulate UPF2-dependent activation of UPF1 in NMD. Structure.

[49] Franks T.M., Singh G., Lykke-Andersen J. (2010). Upf1 ATPase-dependent mRNP disassembly is required for completion of nonsense-mediated mRNA decay. Cell.

[50] Fiorini F., Bagchi D., Le Hir H., Croquette V. (2015). Human Upf1 is a highly processive RNA helicase and translocase with RNP remodelling activities. Nat. Commun..

[51] Huntzinger E., Kashima I., Fauser M., Saulière J., Izaurralde E. (2008). SMG6 is the catalytic endonuclease that cleaves mRNAs containing nonsense codons in metazoan. RNA.

[52] Ohnishi T., Yamashita A., Kashima I., Schell T., Anders K.R., Grimson A., Hachiya T., Hentze M.W., Anderson P., Ohno S. (2003). Phosphorylation of hUPF1 induces formation of mRNA surveillance complexes containing hSMG-5 and hSMG-7. Mol. Cell.

[53] Cho H., Kim K.M., Kim Y.K. (2009). Human proline-rich nuclear receptor coregulatory protein 2 mediates an interaction between mRNA surveillance machinery and decapping complex. Mol. Cell.

[54] Loh B., Jonas S., Izaurralde E. (2013). The SMG5-SMG7 heterodimer directly recruits the CCR4-NOT deadenylase complex to mRNAs containing nonsense codons via interaction with POP2. Genes Dev..

[55] Lejeune F., Li X., Maquat L.E. (2003). Nonsense-mediated mRNA decay in mammalian cells involves decapping, deadenylating, and exonucleolytic activities. Mol. Cell.

[56] Saulière J., Murigneux V., Wang Z., Marquenet E., Barbosa I., Le Tonquèze O., Audic Y., Paillard L., Crollius H.R., Le Hir H. (2012). CLIP-seq of eIF4AIII reveals transcriptome-wide mapping of the human exon junction complex. Nat. Struct. Mol. Biol..

[57] Singh G., Kucukural A., Cenik C., Leszyk J.D., Shaffer S.A., Weng Z., Moore M.J. (2012). The cellular EJC interactome reveals higher-order mRNP structure and an EJC-SR protein nexus. Cell.

[58] Bühler M., Paillusson A., Mühlemann O. (2004). Efficient downregulation of immunoglobulin μ mRNA with premature translation-termination codons requires the 5′-half of the VDJ exon. Nucleic Acids Res..

[59] Carter M.S., Li S., Wilkinson M.F. (1996). A splicing-dependent regulatory mechanism that detects translation signals. EMBO J..

[60] Bühler M., Steiner S., Mohn F., Paillusson A., Mühlemann O. (2006). EJC-independent degradation of nonsense immunoglobulin-μ mRNA depends on 3′ UTR length. Nat. Struct. Mol. Biol..

[61] Lee S.R., Pratt G.A., Martinez F.J., Yeo G.W., Lykke-Andersen J. (2015). Target discrimination in nonsense-mediated mRNA decay requires Upf1 ATPase activity. Mol. Cell.

[62] Ivanov P.V., Gehring N.H., Kunz J.B., Hentze M.W., Kulozik A.E. (2008). Interactions between UPF1, eRFs, PABP and the exon junction complex suggest an integrated model for mammalian NMD pathways. EMBO J..

[63] Silva A.L., Ribeiro P., Inácio Â., Liebhaber S.A., Romão L. (2008). Proximity of the poly(A)-binding protein to a premature termination codon inhibits mammalian nonsense-mediated mRNA decay. RNA.

[64] Tani H., Imamachi N., Salam K.A., Mizutani R., Ijiri K., Irie T., Yada T., Suzuki Y., Akimitsu N. (2012). Identification of hundreds of novel UPF1 target transcripts by direct determination of whole transcriptome stability. RNA Biol..

[65] Hurt J.A., Robertson A.D., Burge C.B. (2013). Global analyses of UPF1 binding and function reveal expanded scope of nonsense-mediated mRNA decay. Genome Res..

[66] Eberle A.B., Stalder L., Mathys H., Orozco R.Z., Mühlemann O. (2008). Posttranscriptional gene regulation by spatial rearrangement of the 3′ untranslated region. PLoS Biol..

[67] Peixeiro I., Inácio Â., Barbosa C., Silva A.L., Liebhaber S.A., Romão L. (2012). Interaction of PABPC1 with the translation initiation complex is critical to the NMD resistance of AUG-proximal nonsense mutations. Nucleic Acids Res..

[68] Wells S.E., Hillner P.E., Vale R.D., Sachs A.B. (1998). Circularization of mRNA by eukaryotic translation initiation factors. Mol. Cell.

[69] Lewis B.P., Green R.E., Brenner S.E. (2003). Evidence for the widespread coupling of alternative splicing and nonsense-mediated mRNA decay in humans. Proc. Natl. Acad. Sci. USA.

[70] Ge Y., Porse B.T. (2014). The functional consequences of intron retention: Alternative splicing coupled to NMD as a regulator of gene expression. BioEssays.

[71] Lareau L.F., Inada M., Green R.E., Wengrod J.C., Brenner S.E. (2007). Unproductive splicing of SR genes associated with highly conserved and ultraconserved DNA elements. Nature.

[72] McGlincy N.J., Tan L.Y., Paul N., Zavolan M., Lilley K.S., Smith C.W.J. (2010). Expression proteomics of UPF1 knockdown in HeLa cells reveals autoregulation of hnRNP A2/B1 mediated by alternative splicing resulting in nonsense-mediated mRNA decay. BCM Genom..

[73] Lareau L.F., Brooks A.N., Soergel D.A.W., Meng Q., Brenner S.E. (2007). The coupling of alternative splicing and nonsense-mediated mRNA decay. Adv. Exp. Med. Biol..

[74] Sureau A., Gattoni R., Dooghe Y., Stévenin J., Soret J. (2001). SC35 autoregulates its expression by promoting splicing events that destabilize its mRNAs. EMBO J..

[75] Sun S., Zhang Z., Sinha R., Karni R., Krainer A.R. (2010). SF2/ASF autoregulation involves multiple layers of post-transcriptional and translational control. Nat. Struct. Mol. Biol..

[76] Rossbach O., Hung L.-H., Schreiner S., Grishina I., Heiner M., Hui J., Bindereif A. (2009). Auto-and cross-regulation of the hnRNP L Proteins by alternative splicing. Mol. Cell. Biol..

[77] Guo J., Jia J., Jia R. (2015). PTBP1 and PTBP2 impaired autoregulation of SRSF3 in cancer cells. Sci. Rep..

[78] Jumaa H., Nielsen P.J. (1997). The splicing factor SRp20 modifies splicing of its own mRNA and ASF/SF2 antagonizes this regulation. EMBO J..

[79] Wollerton M.C., Gooding C., Wagner E.J., Garcia-Blanco M.A., Smith C.W.J. (2004). Autoregulation of polypyrimidine tract binding protein by alternative splicing leading to nonsense-mediated decay. Mol. Cell.

[80] Pervouchine D., Popov Y., Berry A., Borsari B., Frankish A., Guigó R. (2019). Integrative transcriptomic analysis suggests new autoregulatory splicing events coupled with nonsense-mediated mRNA decay. Nucleic Acids Res..

[81] Yang S., Jia R., Bian Z. (2018). SRSF5 functions as a novel oncogenic splicing factor and is upregulated by oncogene SRSF3 in oral squamous cell carcinoma. Biochim. Biophys. Acta Mol. Cell Res..

[82] Wong J.J.L., Ritchie W., Ebner O.A., Selbach M., Wong J.W.H., Huang Y., Gao D., Pinello N., Gonzalez M., Baidya K. (2013). Orchestrated intron retention regulates normal granulocyte differentiation. Cell.

[83] Jones R.B., Wang F., Luo Y., Yu C., Jin C., Suzuki T., Kan M., McKeehan W.L. (2001). The nonsense-mediated decay pathway and mutually exclusive expression of alternatively spliced FGFR2IIIb and-IIIc mRNAs. J. Biol. Chem..

[84] Méreau A., Anquetil V., Lerivray H., Viet J., Schirmer C., Audic Y., Legagneux V., Hardy S., Paillard L. (2015). A posttranscriptional mechanism that controls Ptbp1 abundance in the xenopus epidermis. Mol. Cell. Biol..

[85] McGlincy N.J., Smith C.W.J. (2008). Alternative splicing resulting in nonsense-mediated mRNA decay: What is the meaning of nonsense?. Trends Biochem. Sci..

[86] Winter J., Lehmann T., Krauß S., Trockenbacher A., Kijas Z., Foerster J., Suckow V., Yaspo M.L., Kulozik A., Kalscheuer V. (2004). Regulation of the MID1 protein function is fine-tuned by a complex pattern of alternative splicing. Hum. Genet..

[87] Zheng S., Gray E.E., Chawla G., Porse B.T., O’Dell T.J., Black D.L. (2012). PSD-95 is post-transcriptionally repressed during early neural development by PTBP1 and PTBP2. Nat. Neurosci..

[88] Zheng S. (2016). Alternative splicing and nonsense-mediated mRNA decay enforce neural specific gene expression. Int. J. Dev. Neurosci..

[89] Ni J.Z., Grate L., Donohue J.P., Preston C., Nobida N., O’Brien G., Shiue L., Clark T.A., Blume J.E., Ares M. (2007). Ultraconserved elements are associated with homeostatic control of splicing regulators by alternative splicing and nonsense-mediated decay. Genes Dev..

[90] Änkö M.L., Müller-McNicoll M., Brandl H., Curk T., Gorup C., Henry I., Ule J., Neugebauer K.M. (2012). The RNA-binding landscapes of two SR proteins reveal unique functions and binding to diverse RNA classes. Genome Biol..

[91] Königs V., Machado C.O.F., Arnold B., Blümel N., Solovyeva A., Löbbert S., Schafranek M., Ruiz De Los M.I., Wittig I., McNicoll F. (2020). SRSF7 maintains its homeostasis through the expression of Split-ORFs and nuclear body assembly. Nat. Struct. Mol. Biol..

[92] Saltzman A.L., Kim Y.K., Pan Q., Fagnani M.M., Maquat L.E., Blencowe B.J. (2008). Regulation of multiple core spliceosomal proteins by alternative splicing-coupled nonsense-mediated mRNA decay. Mol. Cell. Biol..

[93] Saltzman A.L., Pan Q., Blencowe B.J. (2011). Regulation of alternative splicing by the core spliceosomal machinery. Genes Dev..

[94] Mitrovich Q.M., Anderson P. (2000). Unproductively spliced ribosomal protein mRNAs are natural targets of mRNA surveillance in C. elegans. Genes Dev..

[95] Cuccurese M., Russo G., Russo A., Pietropaolo C. (2005). Alternative splicing and nonsense-mediated mRNA decay regulate mammalian ribosomal gene expression. Nucleic Acids Res..

[96] Hirschfeld M., Zhang B., Jaeger M., Stamm S., Erbes T., Mayer S., Tong X., Stickeler E. (2014). Hypoxia-dependent mRNA expression pattern of splicing factor YT521 and its impact on oncological important target gene expression. Mol. Carcinog..

[97] Zhou Y., Liu S., Liu G., Öztürk A., Hicks G.G. (2013). ALS-associated FUS mutations result in compromised FUS alternative splicing and autoregulation. PLoS Genet..

[98] Spellman R., Llorian M., Smith C.W.J. (2007). Crossregulation and functional redundancy between the splicing regulator PTB and its paralogs nPTB and ROD1. Mol. Cell.

[99] Hung L.H., Heiner M., Hui J., Schreiner S., Benes V., Bindereif A. (2008). Diverse roles of hnRNP L in mammalian mRNA processing: A combined microarray and RNAi analysis. RNA.

[100] Oberdoerffer S., Moita L.F., Neems D., Freitas R.P., Hacohen N., Rao A. (2008). Regulation of CD45 alternative splicing by heterogeneous ribonucleoprotein, hnRNPLL. Science.

[101] Kemmerer K., Fischer S., Weigand J.E. (2018). Auto-and cross-regulation of the hnRNPs D and DL. RNA.

[102] Änkö M.L., Morales L., Henry I., Beyer A., Neugebauer K.M. (2010). Global analysis reveals SRp20-and SRp75-specific mRNPs in cycling and neural cells. Nat. Struct. Mol. Biol..

[103] Jangi M., Boutz P.L., Paul P., Sharp P.A. (2014). Rbfox2 controls autoregulation in RNA-binding protein networks. Genes Dev..

[104] Valacca C., Bonomi S., Buratti E., Pedrotti S., Baralle F.E., Sette C., Ghigna C., Biamonti G. (2010). Sam68 regulates EMT through alternative splicing-activated nonsense-mediated mRNA decay of the SF2/ASF proto-oncogene. J. Cell Biol..

[105] Levine M. (2010). Transcriptional enhancers in animal development and evolution. Curr. Biol..

[106] Yoshida K., Sanada M., Shiraishi Y., Nowak D., Nagata Y., Yamamoto R., Sato Y., Sato-Otsubo A., Kon A., Nagasaki M. (2011). Frequent pathway mutations of splicing machinery in myelodysplasia. Nature.

[107] Papaemmanuil E., Cazzola M., Boultwood J., Malcovati L., Vyas P., Bowen D., Pellagatti A., Wainscoat J.S., Hellstrom-Lindberg E., Gambacorti-Passerini C. (2011). Somatic SF3B1 mutation in myelodysplasia with ring sideroblasts. N. Engl. J. Med..

[108] Wang L., Lawrence M.S., Wan Y., Stojanov P., Sougnez C., Stevenson K., Werner L., Sivachenko A., DeLuca D.S., Zhang L. (2011). SF3B1 and other novel cancer genes in chronic lymphocytic leukemia. N. Engl. J. Med..

[109] Graubert T.A., Shen D., Ding L., Okeyo-Owuor T., Lunn C.L., Shao J., Krysiak K., Harris C.C., Koboldt D.C., Larson D.E. (2012). Recurrent mutations in the U2AF1 splicing factor in myelodysplastic syndromes. Nat. Genet..

[110] Quesada V., Conde L., Villamor N., Ordóñez G.R., Jares P., Bassaganyas L., Ramsay A.J., Beà S., Pinyol M., Martínez-Trillos A. (2012). Exome sequencing identifies recurrent mutations of the splicing factor SF3B1 gene in chronic lymphocytic leukemia. Nat. Genet..

[111] Rahman M.A., Lin K.T., Bradley R.K., Abdel-Wahab O., Krainer A.R. (2020). Recurrent SRSF2 mutations in MDS affect both splicing and NMD. Genes Dev..

[112] Zhang Z., Krainer A.R. (2004). Involvement of SR proteins in mRNA surveillance. Mol. Cell.

[113] Aznarez I., Nomakuchi T.T., Tetenbaum-Novatt J., Rahman M.A., Fregoso O., Rees H., Krainer A.R. (2018). Mechanism of nonsense-mediated mRNA decay stimulation by splicing factor SRSF1. Cell Rep..

[114] Papaemmanuil E., Gerstung M., Malcovati L., Tauro S., Gundem G., Van Loo P., Yoon C.J., Ellis P., Wedge D.C., Pellagatti A. (2013). Clinical and biological implications of driver mutations in myelodysplastic syndromes. Blood.

[115] Vannucchi A.M., Lasho T.L., Guglielmelli P., Biamonte F., Pardanani A., Pereira A., Finke C., Score J., Gangat N., Mannarelli C. (2013). Mutations and prognosis in primary myelofibrosis. Leukemia.

[116] Kim E., Ilagan J.O., Liang Y., Daubner G.M., Lee S.C.W., Ramakrishnan A., Li Y., Chung Y.R., Micol J.B., Murphy M.E. (2015). SRSF2 mutations contribute to myelodysplasia by mutant-specific effects on exon recognition. Cancer Cell.

[117] Zhang J., Lieu Y.K., Ali A.M., Penson A., Reggio K.S., Rabadan R., Raza A., Mukherjee S., Manley J.L. (2015). Disease-associated mutation in SRSF2 misregulates splicing by altering RNA-binding affinities. Proc. Natl. Acad. Sci. USA.

[118] Varambally S., Dhanasekaran S.M., Zhou M., Barrette T.R., Kumar-Sinha C., Sanda M.G., Ghosh D., Pienta K.J., Sewalt R.G.A.B., Rubin M.A. (2002). The polycomb group protein EZH2 is involved in progression of prostate cancer. Nature.

[119] Bracken A.P., Pasini D., Capra M., Prosperini E., Colli E., Helin K. (2003). EZH2 is downstream of the pRB-E2F pathway, essential for proliferation and amplified in cancer. EMBO J..

[120] Bachmann I.M., Halvorsen O.J., Collett K., Stefansson I.M., Straume O., Haukaas S.A., Salvesen H.B., Otte A.P., Akslen L.A. (2006). EZH2 expression is associated with high proliferation rate and aggressive tumor subgroups in cutaneous melanoma and cancers of the endometrium, prostate, and breast. J. Clin. Oncol..

[121] Kim K.H., Roberts C.W.M. (2016). Targeting EZH2 in cancer. Nat. Med..

[122] Ernst T., Chase A.J., Score J., Hidalgo-Curtis C.E., Bryant C., Jones A.V., Waghorn K., Zoi K., Ross F.M., Reiter A. (2010). Inactivating mutations of the histone methyltransferase gene EZH2 in myeloid disorders. Nat. Genet..

[123] Nikoloski G., Langemeijer S.M.C., Kuiper R.P., Knops R., Massop M., Tönnissen E.R.L.T.M., van der Heijden A., Scheele T.N., Vandenberghe P., de Witte T. (2010). Somatic mutations of the histone methyltransferase gene EZH2 in myelodysplastic syndromes. Nat. Genet..

[124] Sashida G., Harada H., Matsui H., Oshima M., Yui M., Harada Y., Tanaka S., Mochizuki-Kashio M., Wang C., Saraya A. (2014). Ezh2 loss promotes development of myelodysplastic syndrome but attenuates its predisposition to leukaemic transformation. Nat. Commun..

[125] Ghigna C., Giordano S., Shen H., Benvenuto F., Castiglioni F., Comoglio P.M., Green M.R., Riva S., Biamonti G. (2005). Cell motility is controlled by SF2/ASF through alternative splicing of the ron protooncogene. Mol. Cell.

[126] Karni R., de Stanchina E., Lowe S.W., Sinha R., Mu D., Krainer A.R. (2007). The gene encoding the splicing factor SF2/ASF is a proto-oncogene. Nat. Struct. Mol. Biol..

[127] Jia R., Li C., McCoy J.P., Deng C.X., Zheng Z.M. (2010). SRp20 is a proto-oncogene critical for cell proliferation and tumor induction and maintenance. Int. J. Biol. Sci..

[128] Zhou Y.Q., He C., Chen Y.Q., Wang D., Wang M.H. (2003). Altered expression of the RON receptor tyrosine kinase in primary human colorectal adenocarcinomas: Generation of different splicing RON variants and their oncogenic potential. Oncogene.

[129] Ronsin C., Muscatelli F., Mattei M.G., Breathnach R. (1993). A novel putative receptor protein tyrosine kinase of the met family. Oncogene.

[130] Wagh P.K., Peace B.E., Waltz S.E. (2008). Met-related receptor tyrosine kinase ron in tumor growth and metastasis. Adv. Cancer Res..

[131] Krishnaswamy S., Mohammed A.K., Tripathi G., Alokail M.S., Al-Daghri N.M. (2017). Splice variants of the extracellular region of RON receptor tyrosine kinase in lung cancer cell lines identified by PCR and sequencing. BMC Cancer.

[132] Ling Y., Kuang Y., Chen L.L., Lao W.F., Zhu Y.R., Wang L.Q., Wang D. (2017). A novel RON splice variant lacking exon 2 activates the PI3K/ AKT pathway via PTEN phosphorylation in colorectal carcinoma cells. Oncotarget.

[133] He X., Arslan A.D., Pool M.D., Ho T.T., Darcy K.M., Coon J.S., Beck W.T. (2011). Knockdown of splicing factor SRp20 causes apoptosis in ovarian cancer cells and its expression is associated with malignancy of epithelial ovarian cancer. Oncogene.

[134] Iborra S., Hirschfeld M., Jaeger M., Zur Hausen A., Braicu I., Sehouli J., Gitsch G., Stickeler E. (2013). Alterations in expression pattern of splicing factors in epithelial ovarian cancer and its clinical impact. Int. J. Gynecol. Cancer.

[135] Harris A.L. (2002). Hypoxia-a key regulatory factor in tumour growth. Nat. Rev. Cancer.

[136] Chen Y., Du X.Y. (2007). Functional properties and intracellular signaling of CCN1/Cyr61. J. Cell. Biochem..

[137] Mo F.-E., Muntean A.G., Chen C.-C., Stolz D.B., Watkins S.C., Lau L.F. (2002). CYR61 (CCN1) is essential for placental development and vascular integrity. Mol. Cell. Biol..

[138] Wong M., Kireeva M.L., Kolesnikova T.V., Lau L.F. (1997). Cyr61, product of a growth factor-inducible immediate-early gene, regulates chondrogenesis in mouse limb bud mesenchymal cells. Dev. Biol..

[139] Chen C.C., Mo F.E., Lau L.F. (2001). The angiogenic factor Cyr61 activates a genetic program for wound healing in human skin fibroblasts. J. Biol. Chem..

[140] Huang Y.T., Lan Q., Lorusso G., Duffey N., Rüegg C. (2017). The matricellular protein CYR61 promotes breast cancer lung metastasis by facilitating tumor cell extravasation and suppressing anoikis. Oncotarget.

[141] Hirschfeld M., Zur Hausen A., Bettendorf H., Jäger M., Stickeier E. (2009). Alternative splicing of Cyr61 is regulated by hypoxia and significantly changed in breast cancer. Cancer Res..

[142] Zhang B., Zur Hausen A., Orlowska-Volk M., Jäger M., Bettendorf H., Stamm S., Hirschfeld M., Yiqin O., Tong X., Gitsch G. (2010). Alternative splicing-related factor YT521: An independent prognostic factor in endometrial cancer. Int. J. Gynecol. Cancer.

[143] Yamaguchi A., Takanashi K. (2016). FUS interacts with nuclear matrix-associated protein SAFB1 as well as Matrin3 to regulate splicing and ligand-mediated transcription. Sci. Rep..

[144] Tollervey J.R., Curk T., Rogelj B., Briese M., Cereda M., Kayikci M., König J., Hortobágyi T., Nishimura A.L., Župunski V. (2011). Characterizing the RNA targets and position-dependent splicing regulation by TDP-43. Nat. Neurosci..

[145] Qiu H., Lee S., Shang Y., Wang W.Y., Au K.F., Kamiya S., Barmada S.J., Finkbeiner S., Lui H., Carlton C.E. (2014). ALS-associated mutation FUS-R521C causes DNA damage and RNA splicing defects. J. Clin. Investig..

[146] Polymenidou M., Lagier-Tourenne C., Hutt K.R., Huelga S.C., Moran J., Liang T.Y., Ling S.C., Sun E., Wancewicz E., Mazur C. (2011). Long pre-mRNA depletion and RNA missplicing contribute to neuronal vulnerability from loss of TDP-43. Nat. Neurosci..

[147] Jaffrey S.R., Wilkinson M.F. (2018). Nonsense-mediated RNA decay in the brain: Emerging modulator of neural development and disease. Nat. Rev. Neurosci..

[148] Jackson K.L., Dayton R.D., Orchard E.A., Ju S., Ringe D., Petsko G.A., Maquat L.E., Klein R.L. (2015). Preservation of forelimb function by UPF1 gene therapy in a rat model of TDP-43-induced motor paralysis. Gene Ther..

[149] Barmada S.J., Ju S., Arjun A., Batarse A., Archbold H.C., Peisach D., Li X., Zhang Y., Tank E.M.H., Qiu H. (2015). Amelioration of toxicity in neuronal models of amyotrophic lateral sclerosis by hUPF1. Proc. Natl. Acad. Sci. USA..

[150] Udd B., Meola G., Krahe R., Thornton C., Ranum L.P.W., Bassez G., Kress W., Schoser B., Moxley R. (2006). 140th ENMC International Workshop: Myotonic Dystrophy DM2/PROMM and other myotonic dystrophies with guidelines on management. Neuromuscul. Disord..

[151] Kuyumcu-Martinez N.M., Wang G.S., Cooper T.A. (2007). Increased steady-state levels of CUGBP1 in myotonic dystrophy 1 are due to PKC-mediated hyperphosphorylation. Mol. Cell.

[152] Kalsotra A., Wang K., Li P.F., Cooper T.A. (2010). MicroRNAs coordinate an alternative splicing network during mouse postnatal heart development. Genes Dev..

[153] Kalsotra A., Singh R.K., Gurha P., Ward A.J., Creighton C.J., Cooper T.A. (2014). The Mef2 transcription network is disrupted in myotonic dystrophy heart tissue, dramatically altering miRNA and mRNA expression. Cell Rep..

[154] Ho T.H., Bundman D., Armstrong D.L., Cooper T.A. (2005). Transgenic mice expressing CUG-BP1 reproduce splicing mis-regulation observed in myotonic dystrophy. Hum. Mol. Genet..

[155] Ward A.J., Rimer M., Killian J.M., Dowling J.J., Cooper T.A. (2010). CUGBP1 overexpression in mouse skeletal muscle reproduces features of myotonic dystrophy type 1. Hum. Mol. Genet..

[156] Nakamura T., Ohsawa-Yoshida N., Zhao Y., Koebis M., Oana K., Mitsuhashi H., Ishiura S. (2016). Splicing of human chloride channel 1. Biochem. Biophys. Rep..

[157] Mankodi A., Takahashi M.P., Jiang H., Beck C.L., Bowers W.J., Moxley R.T., Cannon S.C., Thornton C.A. (2002). Expanded CUG repeats trigger aberrant splicing of ClC-1 chloride channel pre-mRNA and hyperexcitability of skeletal muscle in myotonic dystrophy. Mol. Cell.

[158] Lueck J.D., Lungu C., Mankodi A., Osborne R.J., Welle S.L., Dirksen R.T., Thornton C.A. (2007). Chloride channelopathy in myotonic dystrophy resulting from loss of posttranscriptional regulation for CLCN1. Am. J. Physiol. Cell Physiol..

[159] Wheeler T.M., Lueck J.D., Swanson M.S., Dirksen R.T., Thornton C.A. (2007). Correction of ClC-1 splicing eliminates chloride channelopathy and myotonia in mouse models of myotonic dystrophy. J. Clin. Investig..

[160] Ding J.H., Xu X., Yang D., Chu P.H., Dalton N.D., Ye Z., Yeakley J.M., Cheng H., Xiao R.P., Ross J. (2004). Dilated cardiomyopathy caused by tissue-specific ablation of SC35 in the heart. EMBO J..

